# Fine-tuned intruder discrimination favors ant parasitoidism

**DOI:** 10.1371/journal.pone.0210739

**Published:** 2019-01-17

**Authors:** Gabriela Pérez-Lachaud, Franklin H. Rocha, Javier Valle-Mora, Yann Hénaut, Jean-Paul Lachaud

**Affiliations:** 1 Conservación de la Biodiversidad, El Colegio de la Frontera Sur, Chetumal, Quintana Roo, Mexico; 2 El Colegio de la Frontera Sur, Tapachula, Chiapas, Mexico; 3 Centre de Recherches sur la Cognition Animale (CRCA), Centre de Biologie Intégrative (CBI), Université de Toulouse; CNRS, UPS, Toulouse, France; Universidade Federal de Uberlândia, BRAZIL

## Abstract

A diversity of arthropods (myrmecophiles) thrives within ant nests, many of them unmolested though some, such as the specialized Eucharitidae parasitoids, may cause direct damage to their hosts. Ants are known to discriminate between nestmates and non-nestmates, but whether they recognize the strength of a threat and their capacity to adjust their behavior accordingly have not been fully explored. We aimed to determine whether *Ectatomma tuberculatum* ants exhibited specific behavioral responses to potential or actual intruders posing different threats to the host colony and to contribute to an understanding of complex ant-eucharitid interactions. Behavioral responses differed significantly according to intruder type. Ants evicted intruders that represented a threat to the colony’s health (dead ants) or were not suitable as prey items (filter paper, eucharitid parasitoid wasps, non myrmecophilous adult weevils), but killed potential prey (weevil larvae, termites). The timing of detection was in accordance with the nature and size of the intruder: corpses (a potential source of contamination) were detected faster than any other intruder and transported to the refuse piles within 15 min. The structure and complexity of behavioral sequences differed among those intruders that were discarded. Workers not only recognized and discriminated between several distinct intruders but also adjusted their behavior to the type of intruder encountered. Our results confirm the previously documented recognition capabilities of *E*. *tuberculatum* workers and reveal a very fine-tuned intruder discrimination response. Colony-level prophylactic and hygienic behavioral responses through effective removal of inedible intruders appears to be the most general and flexible form of defense in ants against a diverse array of intruders. However, this generalized response to both potentially lethal and harmless intruders might have driven the evolution of ant-eucharitid interactions, opening a window for parasitoid attack and allowing adult parasitoid wasps to quickly leave the natal nest unharmed.

## Introduction

Ants are among the most diverse and abundant organisms on earth. Their complex nests and colonies provide both rich, homeostatic microhabitats and available resources that are exploited by other organisms [1–3]. Despite the aggressive behavior and sophisticated defense strategies of most ant species, many organisms (termed in general myrmecophiles) have managed to deal with ant aggressiveness and bypass their defense strategies [2,4–7]. In response, ants have evolved a suite of physiological, immunological and behavioral defensive responses to counter exploitation by micro- and macro-parasites both at the individual and the colony level [8–13].

An efficient system to discriminate between nestmates and non-nestmates is essential not only to maintain the integrity, cohesion and functioning of social insect societies [2], but also to detect intruders and effectively defend the colony [14,15]. Ants are known to effectively discriminate between nestmates and non-nestmates, but their capabilities to detect a threat and adjust their behavioral responses to its intensity have only been addressed recently [16–18].

Nestmate recognition and communication in social insects is based extensively on chemical signals, i.e., cuticular hydrocarbons (CHCs) [19–21], although acoustics also play an important role [22]. Myrmecophiles range from highly integrated guests that rely on physiological, morphological and behavioral adaptations that allow them to be treated as nestmates, to poorly integrated species that elude the hosts as much as possible [23]. Studies on myrmecophiles that exploit ant communication signals to successfully integrate the society are numerous [24,25]. However, very few studies have addressed the processes influencing ant defensive response behaviors against intruders that emerge within the host nest but do not integrate into the colonies [26], which is the case of brood parasitoids and some social parasites. Individual and colony level behavioral defenses represent lower-cost defenses than their physiological counterparts [27], so it is expected that these low-cost defenses be first used when ants encounter an uninvited guest. For example, in bees, only a reduced investment is necessary to express nest sanitation behavior which effectively prevents parasite establishment [28].

Defense at the colony level, also termed “social immunity” [11], encompasses all behaviors that help to prevent invasion by micro- and macro-parasites or infection spread, and includes characteristic sanitation behaviors such as elimination of diseased brood, weeding of infected fungus in leaf-cutter ants, allo-grooming, undertaking (removal of diseased/dead adults; also termed necrophoresis), and waste management among others [11,12,28]. Expressing these behaviors has been shown to effectively impact on the survival and demography of the colony [29]. This is the case particularly with regard to pathogens since social insects are vulnerable to disease transmission due to high density, interaction rate and relatedness of individuals within colonies [5,8,18]. However, how social insects, and in particular ants, combat macro-parasites (arthropods) that threaten their colony resources or their brood has not been fully addressed. It has been suggested that once an alien is inside the nest or emerges within the nest, colony-level prophylactic and hygienic behavioral responses such as active removal of intruders are probably the most general and flexible forms of defense to eliminate them [15,30]. Paradoxically, this general response (removal), well suited to cope with disease [29], might have created a window of evolutionary opportunity for ant parasitoids and might have driven the evolution of ant-parasitoid interactions.

Ants are hosts to at least 17 orders of arthropod myrmecophiles [2,4] spanning from generalist scavengers in the nest (posing no threat to ants and even being beneficial), to very specialized, specific parasitoids and brood predators, which cause direct damage to the colony. Ant parasitoids include representatives of several wasp, fly and mite families [31–33]. Of these, the wasp family Eucharitidae (Hymenoptera) stands out since it is the only family of insects known to exclusively parasitize ants [34,35]. These specific ant parasitoids have a very specialized life cycle [31,34,36]. Females oviposit away from the host upon vegetation; the active searching first instar larvae access the colony through phoresis on foraging ants and develop as ectoparasitoids, initially attaching to ant larvae but completing development when the host pupates [37,38]. Adults emerge inside the host nest among the workers and must escape from the natal nest to mate and reproduce [34,36,38]. Coevolution between ants and these specialized parasitoids is intriguing. It has long been known that ants, in general, do not treat newly emerged eucharitid adults aggressively [39–44], which suggests that both immature and adults may exploit ant communication signals while they are in the nest [42,44]. In a previous study, we experimentally characterized the interactions between freshly eclosed eucharitid parasitoid wasps (*Dilocantha lachaudii* Heraty) and their *Ectatomma tuberculatum* (Olivier) hosts and analyzed their chemical profiles [15]. The results showed that, although partially mimicking the chemical profile of their ant hosts, freshly emerged parasitoids were immediately recognized as alien and removed from the nest [15]. Unscathed wasps were disposed by ants in waste piles outside the nest and could disperse, suggesting that rapid removal of eucharitids by their hosts actually increases the opportunity for these short-lived wasps to complete their life cycle. We also hypothesized that the ant’s general nest hygiene behavior may well be exploited by eucharitids and other ant parasitoids, allowing them to quickly leave the natal nest [15]. However, this previous study left open several questions concerning the specificity of the response of ants: a) whether the observed behavior was specific to eucharitid parasitoids or was a generalized response to any intruder independent of the degree of threat to the colony (i.e., a true general defensive strategy of ants); b) whether removal of eucharitids was comparable to the necrophoric behavior commonly exhibited by ants [1,45,46] as suggested in earlier studies [41]; or c) whether the relatively low aggressiveness of ants was mediated by the calm behavior of the eucharitids (wasps behaving in a steady way, most of the time freezing at contact with an ant), as suggested for other myrmecophiles [47].

In the present study, we carried out an experiment to determine whether ants exhibit differential behavioral responses to intruders that vary in intrinsic characteristics or in the degree of threat to the colony. We aimed at answering the following questions: 1) Do *E*. *tuberculatum* ants remove any alien organism or object from their nests? 2) Do they discriminate among distinct aliens? We further contrasted our results with the behavioral response of ants when encountering eucharitid parasitoid wasps, in an effort to try to disentangle interactions between ants and their specialized parasitoids.

## Materials and methods

### Ethics statement

This study was non-invasive and complied with Mexican law. Collection of insect specimens was authorized by SEMARNAT/DGVS (Permit number Faut-0277 to GP-L). The collection did not involve endangered or protected species. The sites where we collected our species were not protected in any way. Only necessary numbers of *E*. *tuberculatum* were collected to conduct the experiments.

### Study organisms

*Ectatomma tuberculatum* (Formicidae: Ectatomminae) is a Neotropical, generalist, predatory ant, with hypogeic nests built at the base of trees or shrubs. The nest entrance is extended by a chimney made of soil and fragments of vegetable matter [48], which is permanently guarded by specialized workers. Monogyny or facultative polygyny have been reported for different Mexican populations [49,50]. Mature colonies have monomorphic workers with little variation in size [51], but with a well-defined age polyethism in which young workers take care of the brood and then progressively move to foraging tasks while ageing [52,53]. Workers possess a venomous sting and behave aggressively (i.e., repeated stinging and biting) when presented with ants from another colony/species or with potential prey [54].

### Colony collection and maintenance

Four mature, queenright colonies of *E*. *tuberculatum* were studied. Colonies were excavated and collected in January 2013 in Chetumal, Quintana Roo, Mexico (18° 30' 4.54" N; 88° 19' 47.74" W) (S1 Table). The ants were reared in plastic nest boxes (23 x 17 x 10 cm) provided with a glass vial filled with water and stuffed with cotton at one end, and a dry leaf to provide concealment and darkness. Each nest box was connected to a foraging arena (a 30 x 23 x 8 cm, plastic box) via a transparent glass tube (1 cm diam. x 30 cm length). The ants were fed sliced apple, diluted honey and *Tenebrio molitor* L. larvae (Coleoptera: Tenebrionidae) twice a week. Mealworm supply was stopped 3 days prior to and during bioassays. Ants were maintained under laboratory conditions (25–26°C and 50–70% RH and under natural photoperiod) for 4 months before experiments.

### Bioassays

Laboratory tests were conducted using artificially reduced colonies, homogeneous in size with one queen, 50 workers chosen at random from the initial colony (from both the nest and the foraging arena), and the brood. Reduction in colony size does not affect colony functioning [30]. Ant behavior against alien organisms or items inside their nests (hereafter intruders, Table 1) was studied by experimentally placing an intruder in the middle of the nest box. Interactions were video recorded for 15 min beginning immediately after the introduction of the intruder. Observations were performed during May-August 2013, eight to 12 trials were performed per day, between 07:00 and 15:00 h. Treatments were allocated at random to the different colonies (completely randomization design blocked on colony); trials with the same colony were separated by at least 30 min. Fifteen trials per treatment and per colony were conducted for a total of 60 trials per treatment. Intruders were used only once.

**Table 1:** Summary of characteristics of intruders used in this study. Ants were presented with a series of organisms/items providing different multichannel cues that could reveal the stimuli that triggered their behavioral response when encountering an eucharitid eclosing in the nest. These treatments further allowed to test some of the hypotheses that have been put forward to explain ejection of wasps from the natal nest without escalated aggression.

Type of intruder	Intruder characteristics				Level of threat to the colony	Hypothesis tested / treatment
	Chemical profile	Movement	Size (body length) / weight (Mean ± SEM)	Edible/ Inedible		
**Live adult eucharitid wasps (*Dilocantha lachaudii*)**	Imperfect mimic of the ant host chemical profile [15]	Very active insects	3.7 ± 0.05 mm (n = 27); 1.89 ± 0.15 mg [30]	Inedible, hard cuticle	Brood parasitoid, direct damage to the colony	Served as a baseline for ant behavior comparison
**Live *Caulophilus oryzae* adult weevils**	Characterized by [55]	Very active insects	3.1 ± 0.02 mm (n = 20); 1.32 ± 0.04 mg [30]	Inedible, hard cuticle	None	Morphological / structural protection as a means to withstand interactions with ants
**Live *Nasutitermes* sp. workers**	Species-specific mixtures of monoterpenes in their alarm pheromone [56]	Very active insects	4.7 ± 0.05 mm (n = 20)	Common prey	None	Discrimination of prey from intruders (inside the nest)
**Live *C*. *oryzae* larvae**	Larvae possess few CHCs [55]	Apodous, almost motionless	3.2 mm, 1.62 ± 0.44 mg [57]	Potential prey	None	Discrimination of prey from intruders (inside the nest)
**Pentane-washed eucharitids**	Absence of chemical cues (or cues very reduced)	Motionless	same as live eucharitids	Inedible, hard cuticle	None	Chemical insignificance hypothesis [58]
**Dead conspecific workers (killed by freezing, 48 h post-mortem)**	Characteristic oleic and linoleic acids appear post-mortem [59]	Motionless	10.8 ± 0.08 mm (n = 20)	Inedible	High, potential source of microbial contamination	Necrophoresis hypothesis [15,41]
**Small filter paper balls (Whatman #1)**	N/A	Inanimate	1 cm^2^	Inedible	None	Control, neutral object not belonging to the colony (Do ants remove everything from their nests?)

### Treatments

We analyzed ant behavior using a series of objects and live or dead insects (Table 1). Intruders were chosen to investigate four different characteristics that possibly trigger ant responses: mobility, size, whether they are edible, and differences in chemical profile, although these traits cannot be completely disentangled when working with live insects. Six items were tested: (i) motile potential prey (*Nasutitermes* sp. termite workers); (ii) non-motile potential prey (*Caulophilus oryzae* (Gyllenhal) weevil larvae); (iii) live, inedible, small beetles (adult *C*. *oryzae* weevils) as a proxy of live eucharitids due to the possibility of triggering ant behavior through movement detection; (iv) dead, conspecific workers which represent a threat to the colony health; (v) pentane-washed eucharitid wasps, as a proxy of morphological/structural cues provided by eucharitids but in the absence of both chemical cues (no odor or, at least, very reduced signals) and movement; and (vi) filter paper balls as a control. *Ectatomma* ants are known to rely predominantly on visual cues for orientation and foraging [60–63], and are very reactive to movement during predation [51,64]. Consequently, motile or large sized intruders were expected to generate a more rapid response than motionless or smaller ones. Similarly, we hypothesized that dead conspecific ants, representing a threat to the colony’s health, would elicit a more extreme behavioral response from ants than filter paper balls, for example.

*Ectatomma tuberculatum* colonies are parasitized by eucharitids all year round, but generally parasitism rates are very low [50]. Due to the constraints imposed by our experimental design (random allocation of intruders) and because of limited wasp availability, we used recordings from a previous study to characterize behavioral interactions between recently emerged (0–1 day-old) live parasitoids and ants [15]. Recordings were *de novo* visualized and analyzed to produce a flow diagram (see below) which was contrasted against the behavior of ants when confronted with the different intruders tested in this study; such a flow diagram for ants interacting with eucharitids had not been generated before. The bioassays of both our previous and present studies were performed in a similar manner and under similar conditions, but differed in the duration of trials and in the number of workers present in the nests (interactions with live wasps lasted at most 10 min) [15].

Termite workers were collected in the surroundings of Chetumal. They are a common prey for *E*. *tuberculatum* and may constitute up to 9% of their prey in natural field conditions [65]. Larvae and adults of the broad-nosed granary weevil *C*. *oryzae* (Coleoptera: Curculionidae) are currently reared in our laboratory. The hard cuticle of weevil adults might prevent ant attack; furthermore, they are similar in size to eucharitids [30]. Alcohol preserved adult eucharitid wasps (*D*. *lachaudii*) were air dried, soaked in 2 ml GC-grade n-pentane (Fluka Analytical, 99%) for 5 min, and air dried again before use in the bioassays. Conspecific, homo-colonial workers were killed by freezing and then were maintained at room temperature for 48h before trials. Freshly killed *Myrmica rubra* (L.) ants rarely elicit necrophoric behavior in their nestmates while 100% of corpses two-days post mortem do [59]. Filter paper balls were hand-made using pieces of 1 cm^2^ Whatman filter paper and wearing surgical gloves to avoid contamination during manipulation.

### Obtaining data from recordings

Video recordings were independently visualized by two observers. We evaluated the following parameters: latency (the time elapsed between the introduction of the intruder and when the ants initiated a contact), handling plus transport time, and the outcome of interactions. The outcome of the behavioral interactions was scored as: (i) “discarded” (the intruder was removed from the nest or thrown on the interior refusal pile that *E*. *tuberculatum* colonies usually maintain); (ii) “brood provision” (the intruder was stung and injured or killed, then transported to the dry leaf concealing the brood where it was preyed upon; (iii) “ignored” (workers came into contact with the intruder, antennated it without picking it up or transporting it); and (iv) “no decision yet” when ants had not made a clear decision at the end of the observation period (a worker was holding the intruder between its mandibles, and remained still in the middle of the nest). We drew flow diagrams for each intruder type and compared them to the flow diagram obtained using previous recordings of interactions of ants with live wasps. In these figures, 1^st^ order Markov matrices for transition frequencies between two successive behaviors were calculated. Because several ants could participate in interactions with intruders, and the same ant could encounter an intruder more than once during a trial, any trial could include several repetitive short behavioral sequences (loops) ending in item abandon, a behavior referred to as ‘giving- up’ (see below). For each intruder type, percentages were calculated based on the overall number of transitions between individual behavioral acts. All behavioral transitions were represented in the flow diagrams. Primary transitions (those that appeared in at least 3% of the sequences) defined the typical behavioral sequence for a given intruder type.

### Statistical analyses

The outcome of interactions, i.e., the behavioral decision of ants, was analyzed using a logistic regression model with a multinomial response [66,67]. Proportion of intruders that were discarded were compared with Fisher’s exact tests. To determine whether latency and handling plus transport time varied according to the intruder type we used a Generalized Linear Mixed-effects Model (GLMM), with “colony” as a random factor and a binomial error structure fitted to the natural log transformed data [68]. Analyses were performed in R [69]. Data represent mean ± SEM.

## Results

### Outcome of behavioral interactions

**Fig 1:**
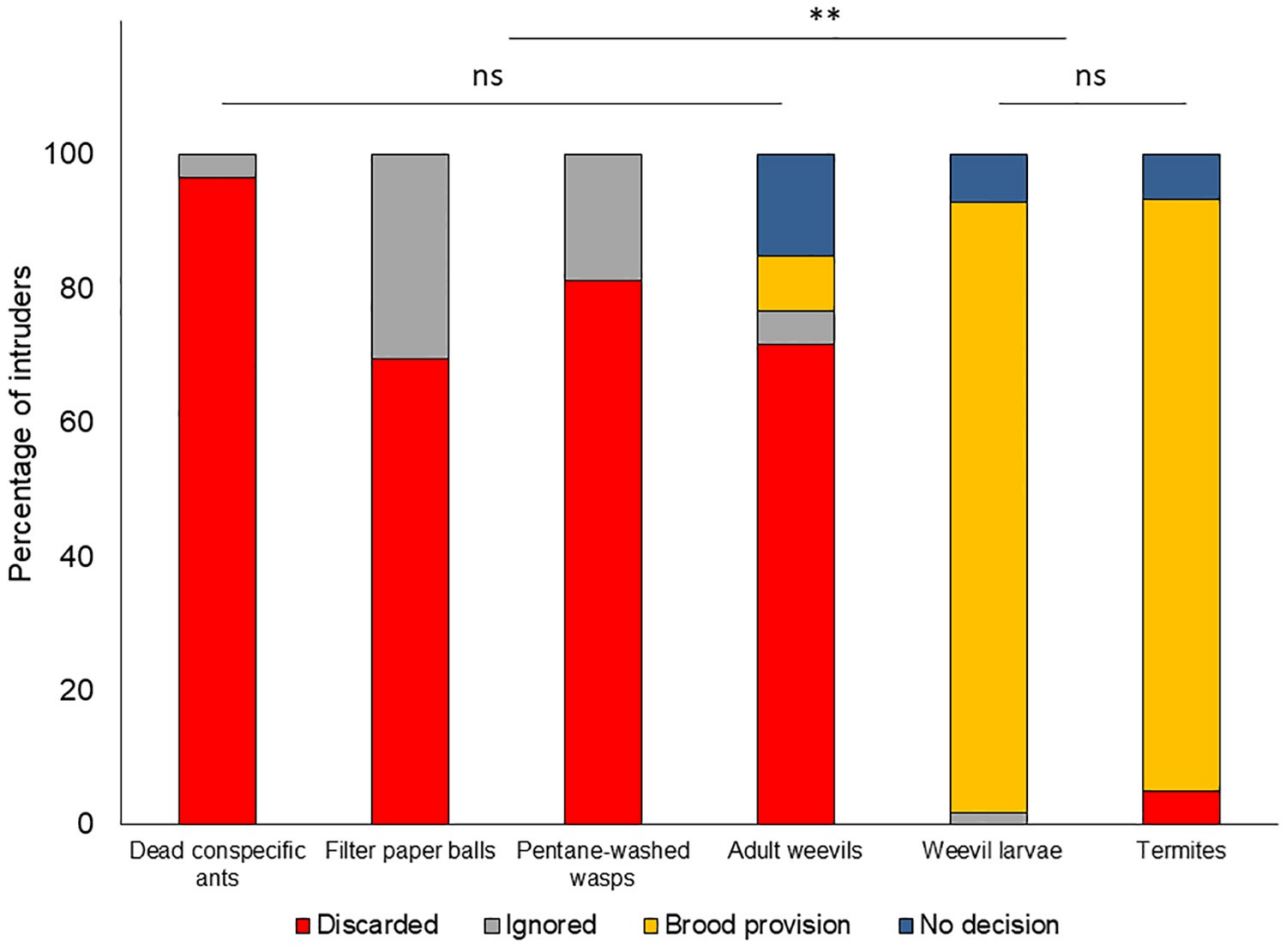
Behavioral response of *Ectatomma tuberculatum* ants against a diverse array of intruders experimentally introduced into their nests. Output of interactions were scored as: “discarded” (the intruder was removed from the nest or deposited in the inner refuse pile in the nest); “brood provisioning” (the intruder was captured and given to the brood as food); “ignored” (the intruder was antennated briefly but remained in its original location at the end of the trial); or “no decision” (no behavioral decision made, the intruder was held between the mandibles of a worker at the end of the trial). Four colonies were tested, n = 60 replicates per treatment.

A total of 360 trials were conducted. Seven trials were excluded from the analyses: 5 recordings were damaged and on two occasions ants did not come into contact with the intruder during the trials. Ants detected the intruder in all 353 remaining observations; of these the intruder was ignored in 35 trials (9.9%), predominantly those with filter paper balls (18 cases) and pentane washed eucharitids (11 cases; Fig 1). In 17 trials (4.8%), the observation period ended while a worker was holding the intruder between its mandibles and no clear decision could be assessed (termites: 4 cases; adult weevils: 9 cases; weevil larvae: 4 cases). Trials with a clear behavioral decision from ants amounted 301. Globally, 89.7% (105/117) of potential prey were effectively preyed upon while 79.7% (188/236) of inedible intruders were discarded during the 15 min observation period.

Ants behavioral response against intruders differed significantly according to the intruder type (logistic regression, *χ*^2^_treatments_ = 411.0, d.f. = 15, p < 0.0001; Fig 1). There were differences among the colonies studied (*χ*^2^_colonies_ = 37.6, d.f. = 9, p < 0.0001), but there was no interaction between these two factors (*χ*^2^_treatments x colonies_ = 35.26, d.f. = 45, p = 0.85). The very efficient nest hygiene response of Colony #1, which did not ignore any intruder, accounted for the differences among colonies (S1 Fig).

Almost all dead conspecific ants were transported to the refuse piles within the 15 min of observation (96.5%, 56/58; 12 to the refuse pile inside the nest and 44 outside). Similarly, pentane-washed wasps, adult weevils and filter paper pieces were, in the vast majority, discarded from the nest (in 81.4, 71.7 and 69.5% of the respective trials). There were no differences among these treatments (Fig 1). By contrast, 91.2% of *C*. *oryzae* larvae and 88.3% of termites were provided to the brood as food (Fig 1). These two last treatments (termites and weevil larvae) were statistically different from the other treatments (95% confidence intervals, S2 Fig). In fact, only the latter two types of intruders (potential prey) were killed or injured; intruders from all other treatments in this study were never injured, and both filter paper balls and corpses did not elicit any aggressive behavior.

The site where ants discarded intruders (refuse pile inside the nest box or outside the nest) was related to intruder type (Pearson´s test of independence, *χ*^2^ = 9.76, d.f. = 3, p < 0.05; Table 2). Filter paper balls were significantly more frequently abandoned in the internal refuse pile than dead ants (Fisher’s exact test, p = 0.044, S2 Table) or adult weevils (Fisher exact test, p = 0.015) which were, in most cases, immediately removed from the nest; pentane-washed wasps were equally transported to both the refuse pile in the nest or outside, and proportions differed only from those of adult weevils. However, all intruders deposited in the interior refuse pile during the observation time were finally removed from the nest one or two hours later.

**Table 2:** Site of removal and percent of individuals discarded by *Ectatomma tuberculatum* workers in 15 min trials. Data from 4 colonies. N is the number of trials in which intruders were discarded.

Treatment	N	Discarded at the interior refuse pile (%)	Removed from the nest (%)
**Dead conspecific ant**	56	21.4	78.6
**Filter paper**	41	41.5	58.5
**Adult weevil**	43	16.3	83.7
**Pentane-washed wasp**	48	37.5	62.5

### Latency and time for handling and transport

**Fig 2:**
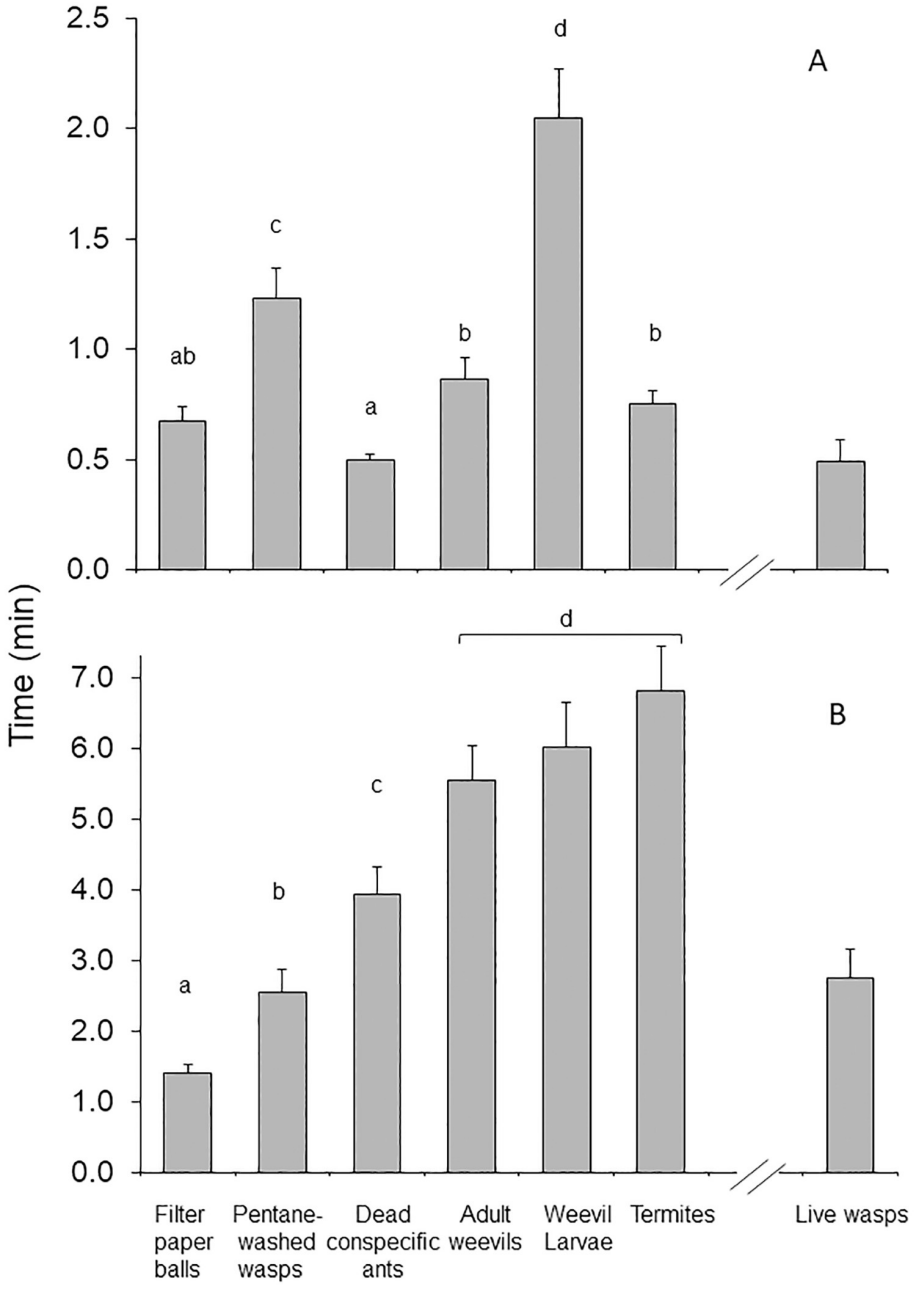
Latency and time for handling and transport of intruders by *Ectatomma tuberculatum* workers. A) Mean (± SEM) latency to contact and B) Mean handling plus transport time. Bioassays were performed with 4 different colonies. Fifteen trials per treatment and per colony were conducted for a total of 60 trials per treatment. Different letters indicate significant differences (p < 0.05). Mean (± SEM) values for live eucharitids from a previous study [15], are shown for reference only, and were not considered in the statistical analyses.

Latency varied according to intruder type (GLMM, *χ*^2^ = 131.46, d.f. = 5, p < 0.001). Dead conspecific workers and filter paper balls were contacted by workers significantly faster (0.50 ± 0.03 min and 0.67 ± 0.06 min after introduction, respectively) than individuals in the other treatments (Fig 2A). Contrastingly, it took longer to detect pentane-washed eucharitids and weevil larvae (1.24 ± 0.14 min and 2.03 ± 0.24 min, respectively), and intermediate latency times characterized trials with termites and adult weevils (0.74 ± 0.06 min and 0.86 ± 0.10 min, respectively) (Fig 2A).

Handling plus transport time also varied according to intruder type (GLMM, *χ*^2^ = 103.65, d.f. = 5, p < 0.001, Fig 2B). Handling and transport of termites and both adult weevils and larvae took significantly longer (more than 5 minutes) compared to the time required to handle dead conspecific ants (3.94 ± 0.39 min), pentane-washed eucharitid wasps (2.55 ± 0.32 min) or filter paper balls (1.34 ± 0.12 min).

### Ant behavioral sequences according to intruder type

Fourteen distinct behavioral acts were involved in interactions with intruders: searching, detection through contact, antennation, mandible strike, seizure-lifting, abdomen bending, stinging attempt, carrying, robbing, failure, giving-up (i.e. abandon of the item after antennation, a behavior that depicts ant motivation), removal, holding motionless and brood provisioning. “Holding motionless” is a behavioral state and refers to a predator subduing a prey and holding it between its mandibles while staying immobile. Cook [48] noticed that when an *E*. *tuberculatum* worker seized a termite, it “held it in its jaws for fully five minutes”. Similarly, workers of this species may remain immobile while holding honey between their mandibles in a behavior known as resource storage [51]. For a detailed description of the rest of the behaviors see [30].

**Fig 3:**
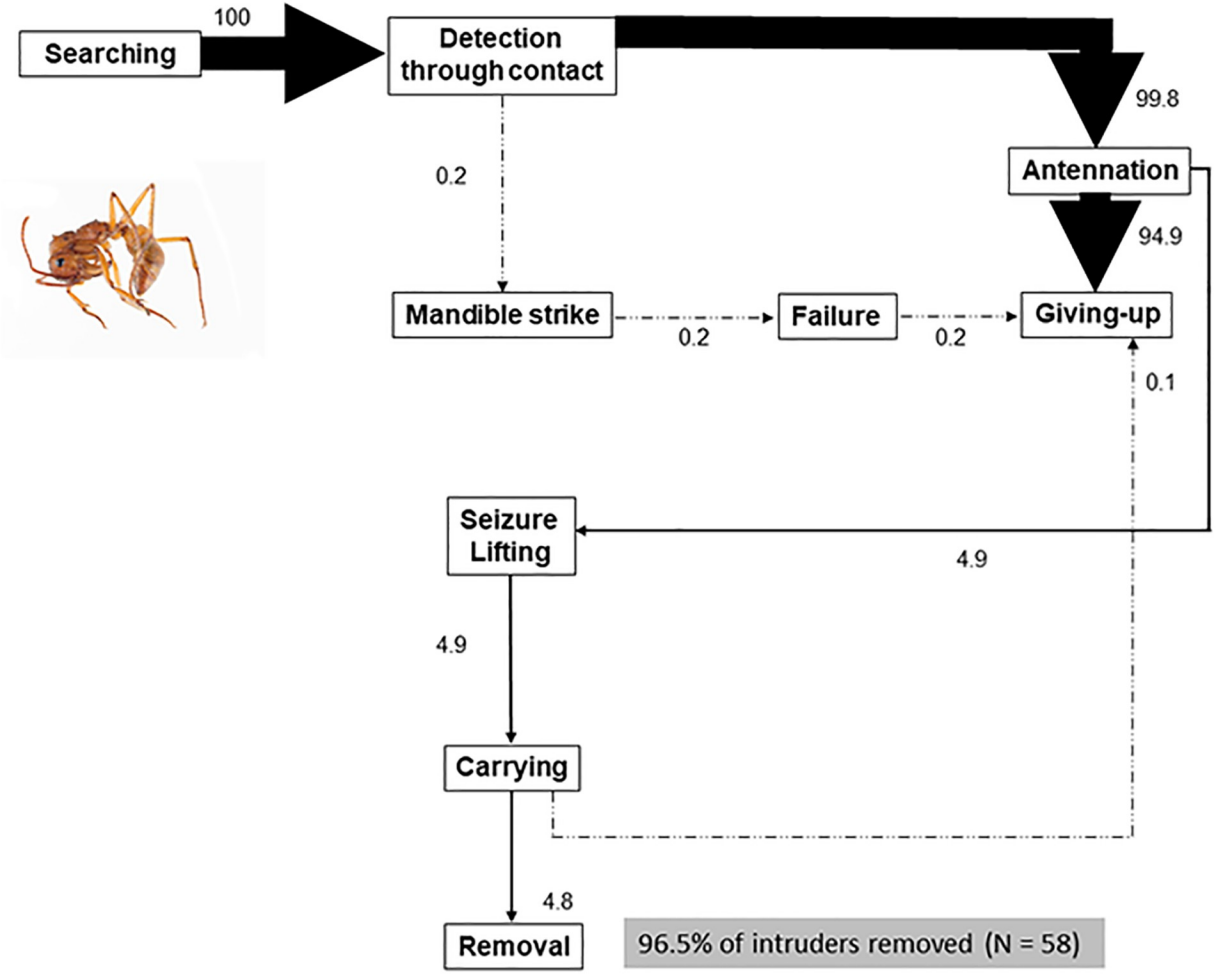
Behavior of *Ectatomma tuberculatum* workers confronted with dead conspecific adult ants. A total of 1168 short incomplete sequences ending in giving-up by the ant, and 56 sequences ending in the removal of the intruder were analyzed (in total: 1224 sequences). The percentage values were calculated as the observed transition frequencies between two successive behavioral acts, divided by the number of sequences. The thickness of each arrow is proportional to the percent value.

The structure and complexity of behavioral sequences differed according to the intruder type. Simple sequences (low number of behavioral acts and few main transitions) were observed with static intruders (ant corpses, pentane-washed wasps and filter paper balls). The behavioral sequence with the simplest (yet the most direct) structure was elicited by corpses of conspecific ants (nine behavioral acts, six major behavioral transitions; Fig 3, Table 3). Corpses elicited high interest and were thoroughly investigated by ants which came repeatedly into contact with them and then departed (20 ± 1.5 contacts per trial; Table 3), promoting numerous new short searching sequences, each one ending in giving-up; however, in 96.5% of the 58 trials the corpse was discarded.

**Table 3:** Summary of characteristics of the behavioral sequences of *E*. *tuberculatum* workers confronted with different types of intruders. Data from 4 colonies; 15 trials per intruder and colony, with the exception of live wasps for which 30 observations were obtained from a previous study [15], but were analyzed here.

Intruder type	Number of trials	Number of sequences analyzed	Number of behavioral acts	Number of main transitions	Mean number of contacts per trial ± SEM (range)
**Dead conspecific ants**	58	1224	9	6	20.6 ± 1.56 (2–54)
**Filter paper balls**	59	617	10	7	10.3 ± 0.81 (1–26)
**Dead, pentane-washed wasps**	59	262	12	13	4.7 ± 0.34 (1–11)
**Live wasps**	28	54	12	18	2.25 ± 0.27 (1–7)
**Live adult weevils**	60	276	12	18	4.7 ± 0.32 (1–12)
**Live termites**	60	786	12	18	13.8 ± 0.91 (1–35)
**Live weevil larvae**	57	213	12	21	3.5 ± 0.23 (1–9)

Trials with filter paper balls and pentane-washed parasitoids involved 10 and 12 behavioral acts, respectively, and few major transitions (Table 3, S3 and S4 Figs). These two types of intruders were mostly gently lifted; however, on a few occasions, and unlike dead ants and filter paper balls, pentane-washed parasitoids were the object of a few mandible strikes and stinging attempts; robbing events between workers also occurred during transport (S4 Fig).

**Fig 4:**
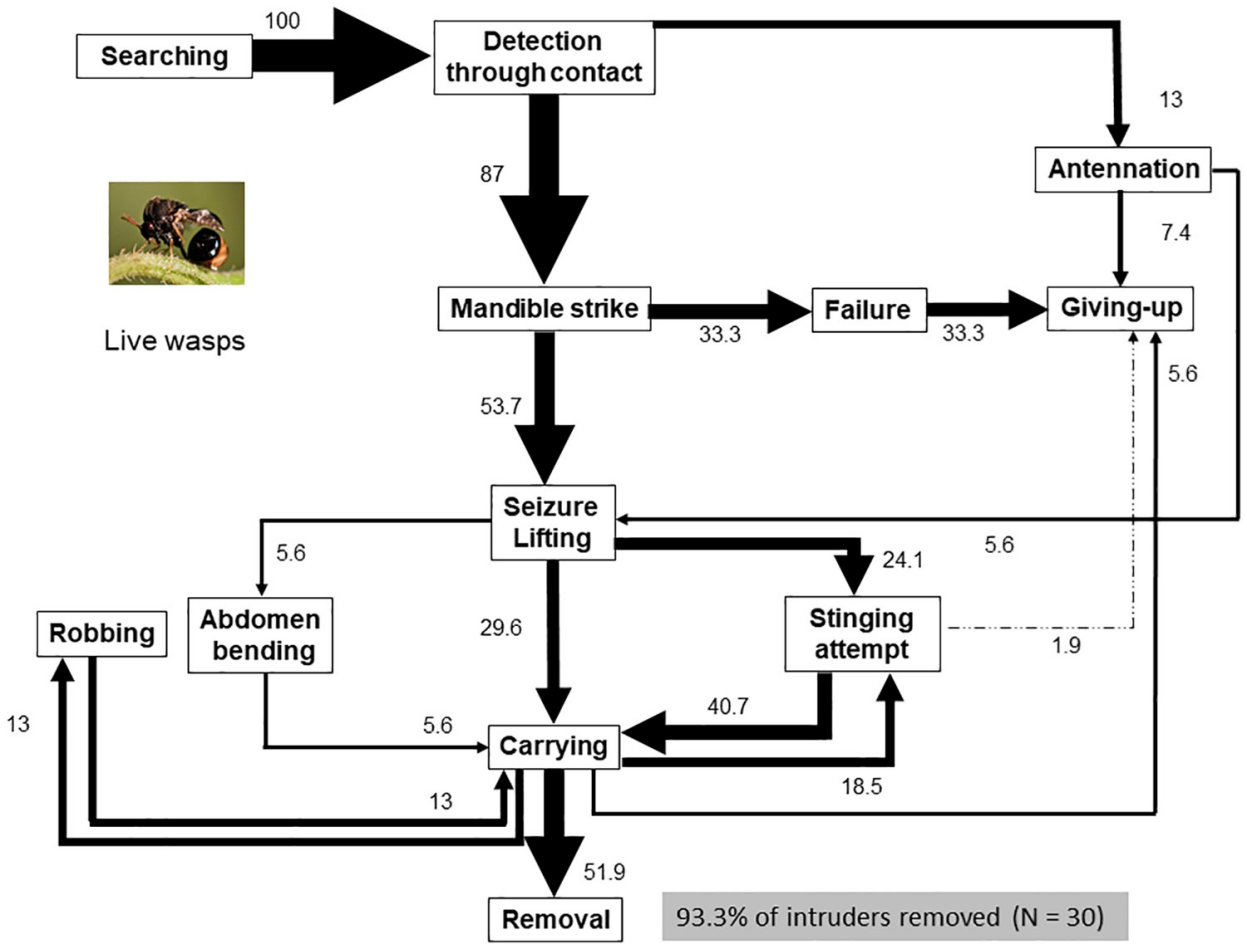
Behavior of *Ectatomma tuberculatum* workers faced with live eucharitid parasitoids. The flow diagram represents a total of 26 incomplete sequences ending in the giving-up by the ant, and 28 complete sequences ending in the removal of the intruder (in total: 54 sequences). Recordings from a previous study [15] were analyzed *de novo*.

Contrastingly, live intruders prompted the expression of the whole defensive/predatory repertoire of ants: behavioral sequences were complex and involved 12 behaviors and between 18 to 21 main transitions (Table 3, Fig 4 and S5–S7 Figs). Failure to catch a live intruder promoted intensive searching once more. Mandibular strike, the sudden closure of mandibles upon the intruder at first contact, was very frequent with live eucharitids (87% of the sequences, Fig 4) and adult weevils (50.7% of the sequences, S5 Fig) while soft-bodied termites and larvae were frequently thoroughly antennated before lifting (S6 and S7 Figs). Stinging attempts (S8 Fig) were provoked by all types of live intruders; however, stinging was only successful with soft bodied termites and weevil larvae in this study. Robbing (a worker fiercely taking the intruder from the transporting worker) was noted with termites (S8 Fig), weevil larvae and live eucharitids, but not with adult weevils (ants did not struggle for transporting weevils). Ants appeared to have some difficulty grabbing adult weevils as, in most trials, ants failed to capture this intruder at the first mandibular strike. Trials with live intruders resulted in high levels of removal and disposal into refuse piles in the cases of live adult weevils (71.7% of the 60 trials) and live wasps (93.3% of the 30 trials, [15]) or in high levels of brood provisioning in the cases of live termites (88.3% of the 60 trials) and live weevil larvae (91.2% of the 57 trials).

Several ants were involved in trials with live insects. Termites were contacted a mean of 13.8 ± 0.9 times, adult weevils and larvae a mean of 4.7 ± 0.3 and 3.5 ± 0.2 times respectively (Table 3), while live parasitoids were, in general, removed by the first or second ant that came into contact with them and were contacted on average 2.2 ± 0.3 times [15]. Workers displayed a frenzied behavior when transporting wasps, which froze and remained still while being transported [15].

## Discussion

Recognition is a basic, major component of biological systems, allowing the distinction of self from non-self and the identification of different classes of non-self [70], and is particularly important in the context of prey/predator discrimination. Like most other social insects, workers of *E*. *tuberculatum* discriminate between nestmates and non-nestmates [54]; they are also able to discriminate their sibling species, the inquiline social parasite *E*. *parasiticum* Feitosa & Fresneau, from their conspecifics [71]. In this study we experimentally demonstrated that *E*. *tuberculatum* workers were, in addition, able to recognize and discriminate among several distinct potential or actual intruders and that they further adjusted their behavior to the type of intruder encountered in the nest. As expected, workers readily discriminated between potential prey and other types of intruders (almost 90% of potential prey were effectively preyed on during the 15 min observation period). This contrasted with the quick removal of the rest of intruders tested here, including those that represented a sanitary risk (dead ants) as well as filter paper, pentane-washed parasitoid wasps, and adult weevils, i.e., all intruders that apparently were of no value as edible items. Similarly, in a previous study [15], inedible live eucharitid parasitoids were quickly removed from the nest. Although there was a high variance in response between the colony fragments tested here, the proportion and type of intruders that were removed/predated were consistent across the four colonies tested. Inter-individual and/or colony personality may explain the observed variability [72,73]. A remarkable consistency in the response of ants was similarly observed in a previous study [15], that included observations on several colonies collected in different years.

Although several different types of intruders elicited the same behavioral response, i.e., removal from the nest, suggesting a general solution to a common problem, trials with intruders that were discarded differed in manner (behavioral acts performed, number of major transitions between behaviors and structure of the behavioral sequence), timing, and location to where the distinct intruders were transported. These differences suggest the existence of high recognition and fine-tuned discrimination capabilities in *E*. *tuberculatum*. Accurate decision-making related to foraging or to a potential threat is crucial for survival, and in social insects this can be achieved either at the individual or colony levels [16,72–74]. Early detection of parasites and/or intruders allows for the initiation of defense mechanisms [18] and is considered crucial to the success of the colony. Fine intruder discrimination abilities have been reported in other social insects, such as bees and stingless bees [75,76]. In *Apis mellifera* L., for example, empirical experimental studies have shown that different invaders elicit different defensive responses in guard bees, from antennation to escalated aggression depending on the threat to the colony [75,77]. In *Temnothorax longispinosus* (Roger) ants, potential enemies (a non-competitor ant species, non-nestmate conspecifics, a competitor species, and a social parasite) are treated differently, based on the threat they inflict on the colony [16]. Furthermore, differential aggressive behavior against myrmecophiles that differ in their degree of integration into the colony has been reported for some ant species such as *Eciton burchellii foreli* Mayr, *Leptogenys processionalis distinguenda* (Emery), *L*. *borneensis* Wheeler, or *Formica rufa* L. [23,78,79].

Our results suggest that both intrinsic characteristics of intruders and the threat posed to ants, influence ant-intruder interactions. As hypothesized, intruder detection by *E*. *tuberculatum* workers was influenced by size and movement: large intruders (ant corpses, filter paper balls) and intruders in movement (adult weevils, termites, live wasps) were detected and contacted significantly earlier than small sized or motionless intruders (pentane-washed eucharitids, weevil larvae), which is consistent with *Ectatomma* ants using predominantly visual cues for orientation and foraging [60–63]. Notably, in our observations, escalated aggression (stinging/killing) was observed only with potential prey, but not (or very infrequently) with other live intruders which were removed unharmed from the nest. Many organisms are known to maximize energy efficiency by engaging in behaviors that are energetically conservative. Since escalated aggression and venom production are costly processes [80,81], accurate recognition of potential prey or judging of potential intruder threats by *E*. *tuberculatum* workers and subsequent adjustment of their behavior in accordance to the threat (escalated aggression or not), can help individuals to avoid wasting time and energy on costly behaviors and hence improve foraging or defense at the colony level.

The response of ants against live adult eucharitids and live adult weevils, small but mobile insects with a hard cuticle, was quite similar, and though interactions were in most cases not fatal, mandible strikes and stinging attempts were frequently employed with these two intruders, demonstrating a certain level of aggressiveness. Sequences with weevils and eucharitids differed though in the interest that these two intruders elicited in ants and the eagerness of ants to get rid of eucharitids (frenzied behavior of ants and robbing of the intruder between nestmates was observed with live eucharitids but not with adult weevils), which might be explained by differences in their CHC profiles. By contrast, the response of ants to pentane-washed wasps (likely bearing few or no CHCs) was characterized by prolonged antennation of the static wasp and the virtual absence of aggressive behaviors. Absence of or reduced aggression of ants against non-nestmates and some myrmecophiles is thought to correlate with a reduction in chemical cues (chemical insignificance hypothesis; [58]). This hypothesis might explain the low aggressiveness of ants against live eucharitids but under closer examination does not explain their removal from the nest. Furthermore, it appears that intruder recognition in *E*. *tuberculatum* does not rely exclusively on chemical cues because items such as pentane-washed wasps and filter paper balls were also removed from the nest. Significantly, the response of ants with pentane-washed wasps, filter paper balls, and dead conspecific ants was characterized by prolonged antennation and the gentle seizure of the intruder. These three types of immobile intruders bearing distinct chemical cues triggered a thorough exploration in the ants but no aggression, suggesting a role for movement in eliciting aggression. Motion, supplemented perhaps with chemical cues, may release aggressive behaviors such as mandible strike and stinging attempts and may promote the willingness of ants to remove live wasps.

Taken together, our observations suggest that inedible intruders are systematically removed from the nest, no matter their nature, and indicate the combination of at least three intruder traits affecting the expression of individual ant behavior during interactions which could explain the relatively low aggressive behavior of ants against the parasitoid wasps: a) morphological/structural defenses of eucharitids and other similar intruders (tough cuticle) that hampers ant attack, b) thanatosis and calm behavior or immobility of the intruder, and c) tiny size of the intruder relative to ants (see Table 1 and S8 Fig). In trials with adult weevils, stinging attempts were numerous but unsuccessful; the morphology and hard cuticle of weevils were apparently an impediment to achieve attack. Likewise, stinging attempts were in most cases unsuccessful with live eucharitids, and escalated attacks (sustained stinging attempts until wasp death) have been observed only twice as part of another study [15].

Members of Eucharitidae are all ant parasitoids and their interaction with ants is shaped by a long co-evolutionary history, with eucharitids colonizing ants approximately 72 My ago [35]. Members of the tribe Eucharitini exhibit an extraordinary amount of morphological variation, particularly concerning the thoracic spines (from cylindrical to carapace-like [35], see also Fig. 26 in [82] and Fig 6 in [83]) that usually cover the gaster, the part of the body most susceptible to physical harm (GP-L, pers. obs.). A protective role of these posterior scutellar processes during interactions with aggressive ants has already been suggested [82], and our results with eucharitids and adult weevils add further support for such a protective role of both the hard cuticle of some insects and the thoracic spines of Eucharitini. Analogous structural defenses have been reported in various other myrmecophiles that exhibit different life styles. For example, a hard exoskeleton allows the brood predator beetle *Myrmecaphodius excavaticollis* (Blanchard) to withstand initial interactions with *Solenopsis invicta* Buren workers before acquiring the host colony odor [84]; while the hard but smooth cuticle of the adults of the mesostigmatid parasitoid mite *Macrodynichus sellnicki* Hirschmann & Zirngiebl-Nicol thwarts *Nylanderia fulva* (Mayr) ants from grasping them, allowing the mites to remain in the nest and attack the brood [85].

As already noted, and as for several other ant species [86–89], our results suggest an important role of movement in triggering stinging in *E*. *tuberculatum* workers. This indicates that the freezing and calm behavior of live eucharitids is responsible for the relatively low aggressiveness frequently reported in the literature in ant-eucharitid interactions [39–44], and invoked in other myrmecophiles to explain ant tolerance [47]. Static, inedible intruders in this study (pentane-washed wasps, dead ants and filter paper balls) were not treated aggressively, though ants did discriminate among them. Significantly, dead ants elicited a stronger response than the other two static intruders (many more ants came into contact with dead ants that were antennated more frequently than filter paper balls or pentane-washed wasps) and elicited the most direct (in terms of sequence structure) and fastest response in ants. This is consistent with the rapid disposal of corpses documented for other ant species. It is known that oleic acid, a compound produced by the decomposition of corpses, is a strong stimulus that releases necrophoric behavior in ants [29,90]. Necrophoresis and refuse disposal have been previously advanced as plausible explanations of eucharitid removal from the host nest [15,41]. However, a detailed comparison of the flow diagrams between live eucharitids and corpses (Figs 3 and 4) did not support the necrophoresis hypothesis as an explanation of live eucharitid transport and removal from the natal nest.

Incidentally, the relative small size of eucharitids, reinforced by their calm behavior, may contribute to reduced ant aggressiveness. Adult wasps are small relative to *Ectatomma* workers (S8 Fig) and though they are recognized as alien, they might not be perceived as a real threat [15]. Similarly, low aggression of the host was also noted for two species of *Macrodinychus* mites which are only 2 mm in length and therefore small when compared with their *Leptogenys* hosts: in artificial nests, mites were frequently picked up and dumped at the nest refuse site [26].

Our final aim was to try to elucidate the stimuli that promote wasp removal without aggression. On the whole, *E*. *tuberculatum* workers removed most of the intruders from their nests, with the exclusion of potential prey, illustrating a very efficient nest hygiene behavior which may well have had consequences for the evolution of ant parasitoidism. Very few studies have examined the interactions with intruders that develop inside the nest during a stage that is “invisible” to ants before emerging and attempting to leave the natal site to continue with their life cycle (e.g. offspring of social parasites and parasitoids that emerge inside the host colonies). It is known, for example, that male offspring of the bumblebee social parasite, *Bombus vestalis vestalis* (Geoffroy), which actively produces sexual pheromones, uses repellent substances to escape from host aggression during their short intranidal life [14]. However, to our knowledge, eucharitid parasitoids do not employ such a strategy and no appeasement substances have been reported in these wasps [42]. Rapid escape from the natal nest is important to the wasps’ overall fitness because they are very short-lived, and exploitation of the predictable hygiene nest response of ants (systematic removal) by eucharitids, represents a quick means of leaving the host nest [15]. The generalization of the removal response to adult weevils in this study seems to support the exploitation of the ant hygienic response hypothesis by *D*. *lachaudii*, and also by a number of other ant parasitoids.

Despite the range of potential or actual intruders used in our experiment, *E*. *tuberculatum* workers behaved in a very stereotyped and specific way according to the type of intruder encountered within their nests, exhibiting a very flexible response which is congruent with both their very flexible behavior during foraging [62] and their learning capabilities [63, 91]. Our results showed an adjustment of their behavior to the characteristics of the intruder, with a heightened defensive response towards parasitoids and adult weevils and a rapid removal of corpses, but no aggression toward invaders that did not represent a threat to the colony. This behavioral flexibility may have an adaptive value in the field when ants have to deal with a variety of natural enemies such as parasitoids or conspecific and heterospecific alien ants that intrude into the nests to steal food, for example [92–94]. From an adaptive perspective, the more generalized but flexible the defensive response, the more readily the colonies adjust to potential threats.

## Supporting information

S1 Fig Colony related effect upon the behavioral response of *Ectatomma tuberculatum* ants against a diverse array of intruders experimentally introduced into their nests. Colony 1 showed the most efficient nest hygienic behavior: the probability of an intruder being ignored was null.
(TIF)

S2 Fig Effect of treatment (intruder type) upon the behavioral response of *Ectatomma tuberculatum* ants. Termites and weevil larvae treatments differed from the rest of the treatments.
(TIF)

S3 Fig Behavior of *Ectatomma tuberculatum* workers with filter paper balls. A total of 575 short incomplete sequences and 42 sequences ending in the removal of the filter paper were analyzed (in total: 617 sequences).
(TIF)

S4 Fig Behavior of *Ectatomma tuberculatum* workers faced with dead, pentane-washed, eucharitid parasitoids. In most trials initial inspection of the intruder ended rapidly after a brief contact with the wasp. A total of 213 incomplete and 49 complete sequences were analyzed (in total: 262 sequences).
(TIF)

S5 Fig Behavior of *Ectatomma tuberculatum* workers faced with live adult *Caulophilus oryzae* weevils. A total of 222 incomplete, 37 complete and 17 sequences that ended with the weevil being held by a worker were analyzed (in total: 276 sequences).
(TIF)

S6 Fig Behavioral interactions of *Ectatomma tuberculatum* workers with live termites. A total of 729 incomplete sequences ending in the giving-up of the ant, 53 complete sequences ending with the termite being provided to the brood, and 4 sequences ending with the termite held by a worker were analyzed (in total: 786 sequences).
(TIF)

S7 Fig Behavior of *Ectatomma tuberculatum* workers faced with live *Caulophilus oryzae* larvae. A total of 157 incomplete sequences, 52 complete sequences ending with the intruder transported to the brood, and 4 sequences ending with the intruder being held between the mandibles of a worker were analyzed (in total: 213 sequences).
(TIF)

S8 Fig Behavior of *Ectatomma tuberculatum* workers during interactions with live intruders. A) Stinging attempt against an adult weevil; B) Robbing (a worker fiercely taking a termite transported by a nestmate); C) Transport of a live eucharitid; D) Transport of a live adult weevil. Background of A, B and D is 1 mm square graph paper.
(TIF)

S1 Table Composition of *Ectatomma tuberculatum* colonies. Colonies were excavated and collected in January 2013 in Chetumal, Quintana Roo, Mexico (18° 30' 4.54" N; 88° 19' 47.74" W).
(TIF)

S2 Table Pairwise comparisons of proportion of intruders directly removed from the nest and those transported to the interior refuse pile. Fisher’s exact test probabilities; significantly different values in bold.
(TIF)

S3 Table Latency, handling and transport time, outcome of interactions, and size of intruders and *E*. *tuberculatum* workers. (XLSX)
