# Supplementary figures and images for: Fine-tuned intruder discrimination favors ant parasitoidism
Source: PLoS One. 2019 Jan 17;14(1):e0210739. doi: 10.1371/journal.pone.0210739 (PMC6336292; doi:10.1371/journal.pone.0210739)

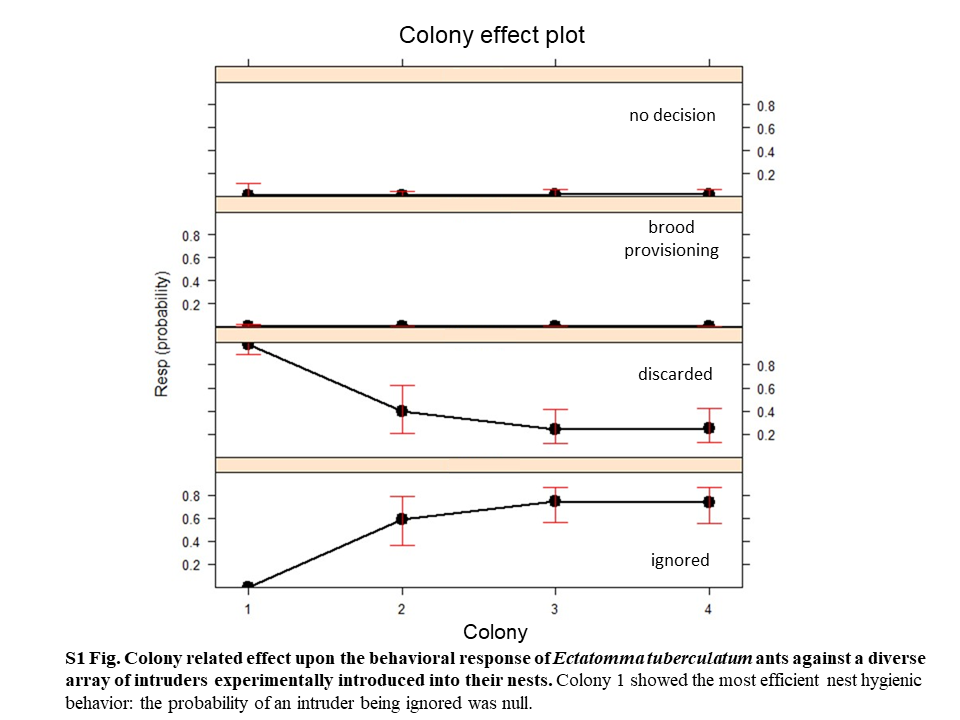

Supplement: S1 Fig — Colony 1 showed the most efficient nest hygienic behavior: the probability of an intruder being ignored was null. (TIF) [file pone.0210739.s001.tif]

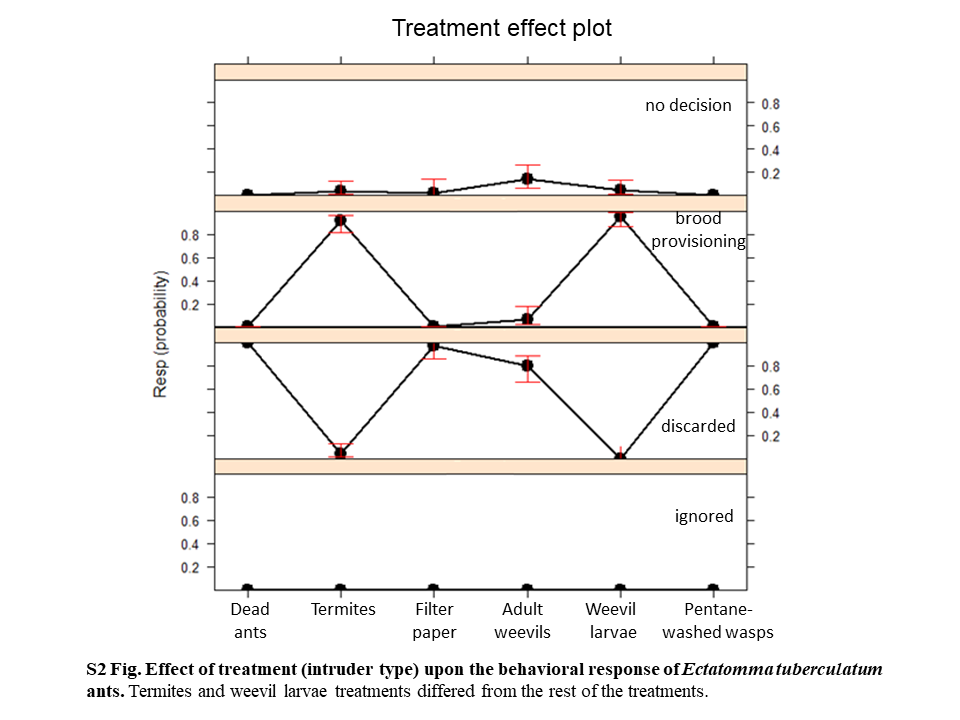

Supplement: S2 Fig — Termites and weevil larvae treatments differed from the rest of the treatments. (TIF) [file pone.0210739.s002.tif]

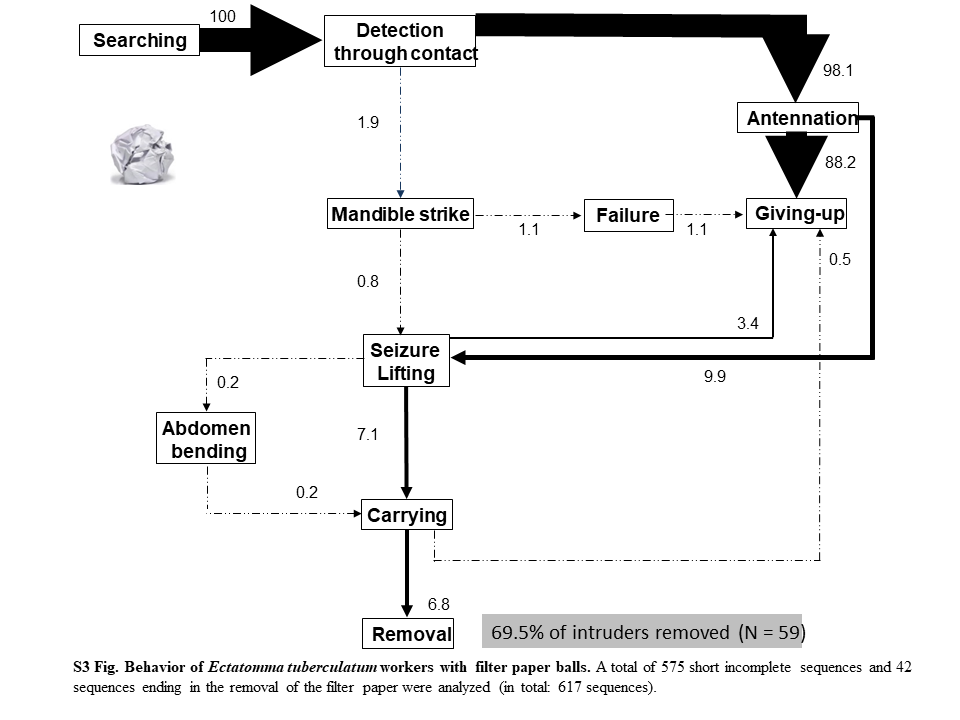

Supplement: S3 Fig — A total of 575 short incomplete sequences and 42 sequences ending in the removal of the filter paper were analyzed (in total: 617 sequences). (TIF) [file pone.0210739.s003.tif]

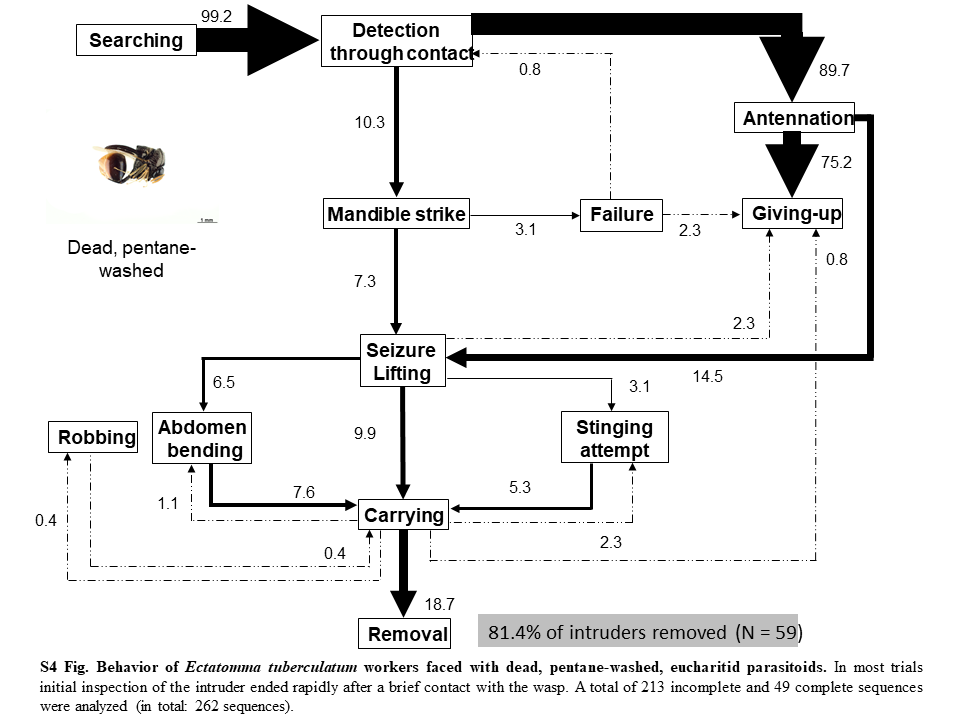

Supplement: S4 Fig — In most trials initial inspection of the intruder ended rapidly after a brief contact with the wasp. A total of 213 incomplete and 49 complete sequences were analyzed (in total: 262 sequences). (TIF) [file pone.0210739.s004.tif]

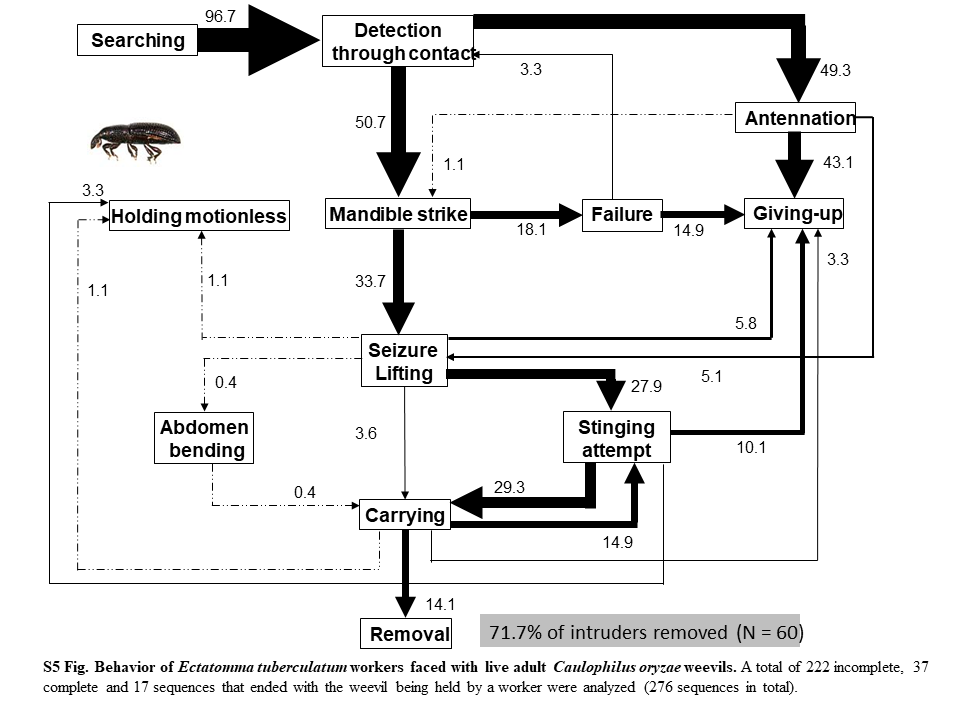

Supplement: S5 Fig — A total of 222 incomplete, 37 complete and 17 sequences that ended with the weevil being held by a worker were analyzed (in total: 276 sequences). (TIF) [file pone.0210739.s005.tif]

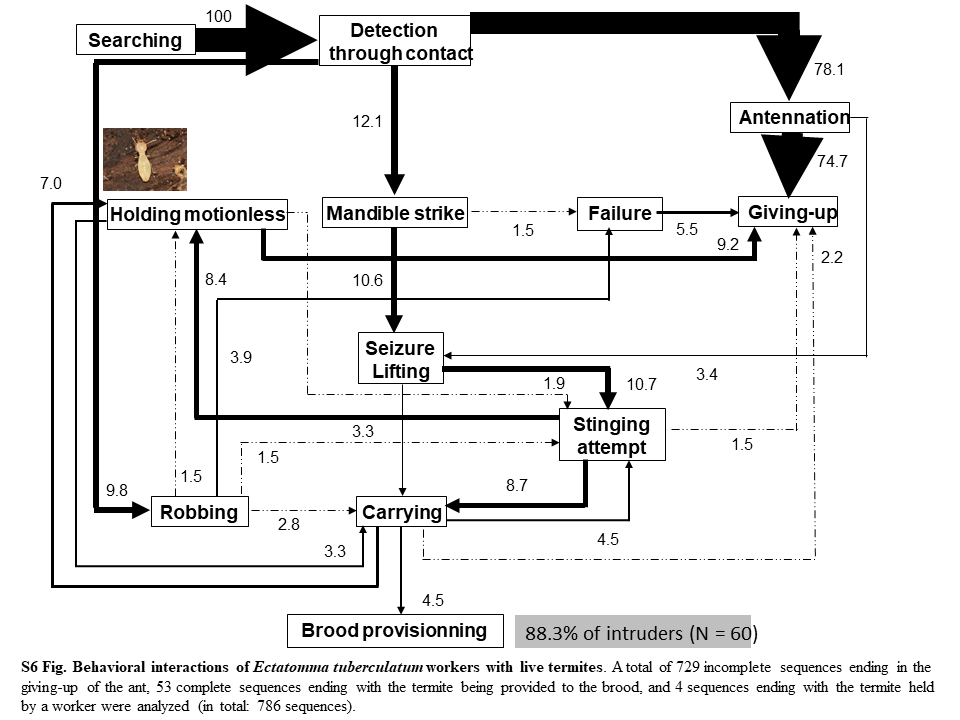

Supplement: S6 Fig — A total of 729 incomplete sequences ending in the giving-up of the ant, 53 complete sequences ending with the termite being provided to the brood, and 4 sequences ending with the termite held by a worker were analyzed (in total: 786 sequences). (TIF) [file pone.0210739.s006.tif]

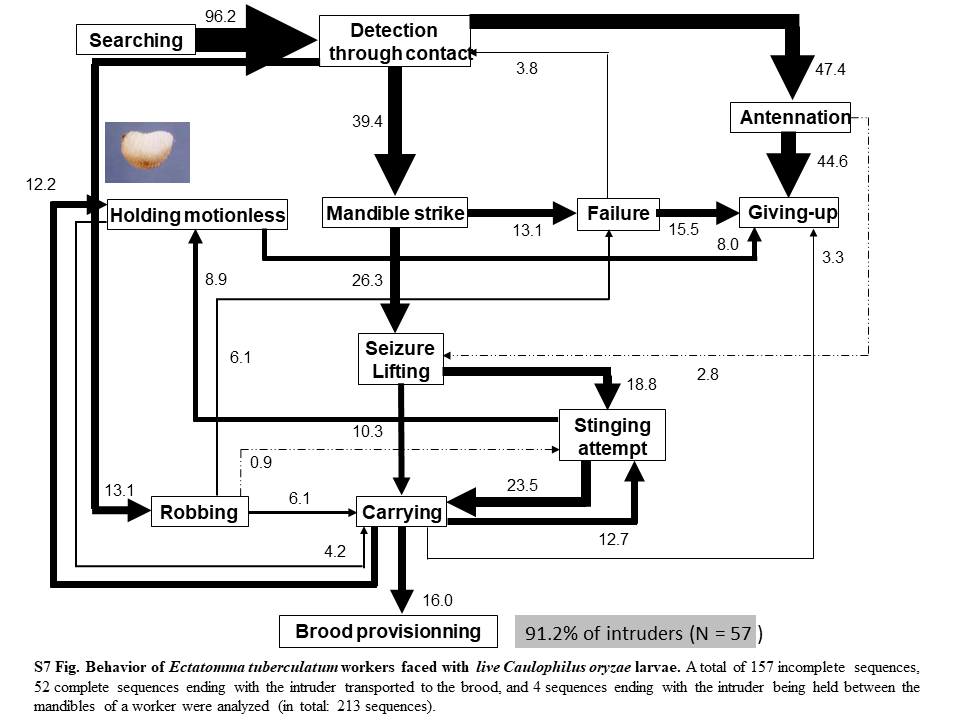

Supplement: S7 Fig — A total of 157 incomplete sequences, 52 complete sequences ending with the intruder transported to the brood, and 4 sequences ending with the intruder being held between the mandibles of a worker were analyzed (in total: 213 sequences). (TIF) [file pone.0210739.s007.tif]

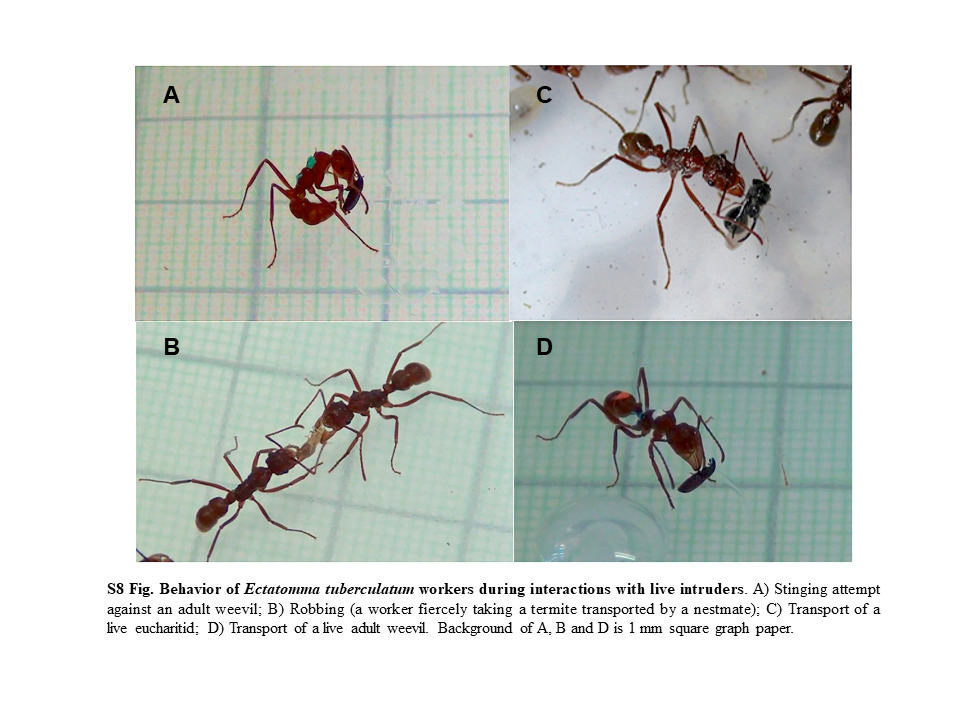

Supplement: S8 Fig — A) Stinging attempt against an adult weevil; B) Robbing (a worker fiercely taking a termite transported by a nestmate); C) Transport of a live eucharitid; D) Transport of a live adult weevil. Background of A, B and D is 1 mm square graph paper. (TIF) [file pone.0210739.s008.tif]

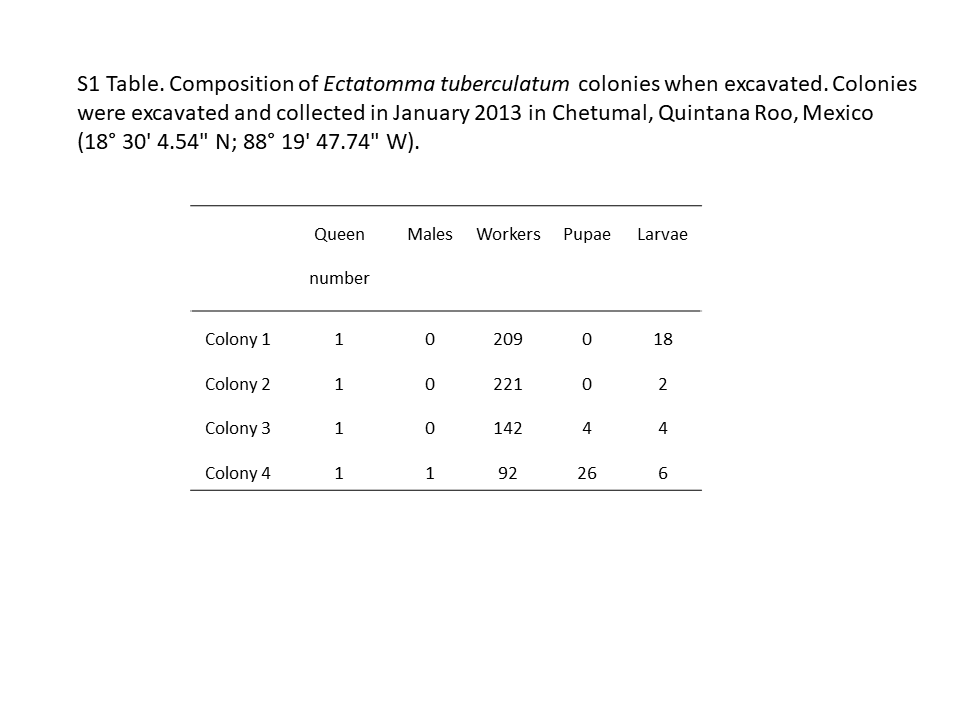

Supplement: S1 Table — Colonies were excavated and collected in January 2013 in Chetumal, Quintana Roo, Mexico (18° 30' 4.54" N; 88° 19' 47.74" W). (TIF) [file pone.0210739.s009.tif]

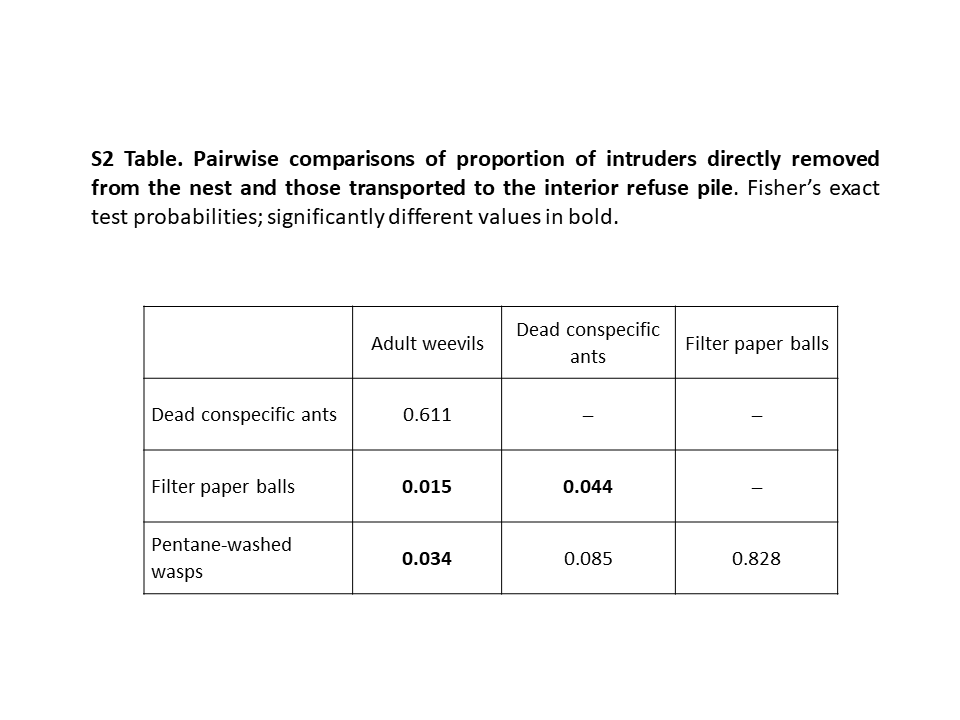

Supplement: S2 Table — Fisher’s exact test probabilities; significantly different values in bold. (TIF) [file pone.0210739.s010.tif]
